# Awakening: Predicting external stimulation to force transitions between different brain states

**DOI:** 10.1073/pnas.1905534116

**Published:** 2019-08-19

**Authors:** Gustavo Deco, Josephine Cruzat, Joana Cabral, Enzo Tagliazucchi, Helmut Laufs, Nikos K. Logothetis, Morten L. Kringelbach

**Affiliations:** ^a^Center for Brain and Cognition, Computational Neuroscience Group, Universitat Pompeu Fabra, 08018 Barcelona, Spain;; ^b^Department of Information and Communication Technologies, Universitat Pompeu Fabra, 08018 Barcelona, Spain;; ^c^Institució Catalana de la Recerca i Estudis Avançats, 08010 Barcelona, Spain;; ^d^Department of Neuropsychology, Max Planck Institute for Human Cognitive and Brain Sciences, 04103 Leipzig, Germany;; ^e^Turner Institute for Brain and Mental Health, Monash University, Melbourne, Clayton, VIC 3800, Australia;; ^f^Department of Psychiatry, University of Oxford, Oxford OX3 7JX, United Kingdom;; ^g^Center for Music in the Brain, Department of Clinical Medicine, Aarhus University, 8000 Aarhus, Denmark;; ^h^Life and Health Sciences Research Institute, School of Medicine, University of Minho, 4710-057 Braga, Portugal;; ^i^Institute for Medical Psychology, Christian Albrechts University, 24118 Kiel, Germany;; ^j^Department of Neurology and Brain Imaging Center, Goethe University, 60528 Frankfurt am Main, Germany;; ^k^Department of Neurology, Christian Albrechts University, 24118 Kiel, Germany;; ^l^Department Neurophysiology of Cognitive Processes, Max Planck Institute for Biological Cybernetics, 72076 Tübingen, Germany;; ^m^Imaging Science and Biomedical Engineering, University of Manchester, Manchester M13 9PT, United Kingdom

**Keywords:** brain states, metastates, electrical stimulation, computational neuroscience, modeling

## Abstract

A fundamental problem in systems neuroscience is how to force a transition from one brain state to another by external driven stimulation in, for example, wakefulness, sleep, coma, or neuropsychiatric diseases. This requires a quantitative and robust definition of a brain state, which has so far proven elusive. Here, we provide such a definition, which, together with whole-brain modeling, permits the systematic study in silico of how simulated brain stimulation can force transitions between different brain states in humans. Specifically, we use a unique neuroimaging dataset of human sleep to systematically investigate where to stimulate the brain to force an awakening of the human sleeping brain and vice versa. We show where this is possible using a definition of a brain state as an ensemble of “metastable substates,” each with a probabilistic stability and occurrence frequency fitted by a generative whole-brain model, fine-tuned on the basis of the effective connectivity. Given the biophysical limitations of direct electrical stimulation (DES) of microcircuits, this opens exciting possibilities for discovering stimulation targets and selecting connectivity patterns that can ensure propagation of DES-induced neural excitation, potentially making it possible to create awakenings from complex cases of brain injury.

## Significance

We describe a quantitative and robust definition of a brain state as an ensemble of “metastable substates,” each with a probabilistic stability and occurrence frequency. Fitting this to a generative whole-brain model provides an innovative avenue for predicting where simulated brain stimulation can force transitions between different brain states. We provide proof-of-concept by systematically applying this model framework to neuroimaging data of the human sleep cycle and show where to stimulate to awaken the human sleeping brain and vice versa. These results suggest an avenue for using causal whole-brain models to discover in silico where to force a transition between brain states, which may potentially support recovery in disease.

Almost 13 decades ago, the father of cognitive psychology, William James, wrote “everybody knows what attention is” (1). Indeed, we have long been recognizing various “states of the brain,” including sleep, wakefulness, aphasia, or attention, but our understanding of the actual “brain states” underlying such states remains poor, to say the least. Brains have billions of neurons and trillions of synapses, nested recursive circuits at all possible spatiotemporal levels, massive connectivity, and initial condition-dependent activity evolution. In disciplines such as physics, genomics, or economics, such systems are characterized as complex dynamic ones, whereby complex implies that the ultimately emerging global behavior cannot be understood by merely studying the network’s elementary nodes. The continuously evolving dynamics of such widespread networks are characterized by a condition-dependent self-organization, going through stable, “quasistable,” high or low activities and transient arrangements, termed brain states. Given the complexity of brain states and their probabilistic, often chaotic, state transitions, their in-depth study up to now has been minimally successful.

Still, attempts have been made to reduce this complexity to meaningful insights, e.g., by defining a state space in human neuroimaging data, using, for example, an estimation of the relationship of selected spatiotemporal and spectral characteristics of activity within a window shifted over time. The high dimensionality of signals in such cases can be reduced to 2 or 3 values plotted in a low-dimensional state space enabling visualization and easier interpretation of the data (2–5). However, while this approach has been very successful in making sense of complex data, such a representation ultimately fails to capture the important dynamics of states and their transitions.

Drawing on insights from complex systems, brain states can also be described as attractors, i.e., stable states of interacting brain regions, offering insights into repeatable and robust system configurations (5, 6), or by additionally using generative models that take into account context- and rank-dependent constraints at different hierarchical processing levels (7–9). Interregional interactions are commonly reflected in the degree of coupling of neural activity oscillations of local (microcircuit) or remote neuromodulatory origin.

All of these approaches, however, fail to capture the concept of metastability, i.e., the quality of systems, including the brain, to temporarily persist in an existing equilibrium despite slight perturbations. Evidently, the dynamics of coordination between brain regions with high functional differentiation is less likely to be as stable and persistent as the coupling observed between, say, areas that are part of a single sensory or motor system. The coordination patterns within stable states may often reflect dynamically recurring short-lived patterns that can be clustered in metastable substates for any given brain state (see, e.g., ref. 10).

In the current study, we provide a definition of brain states dubbed “characterization of the probabilistic metastable substates (PMS) space,” which fully typifies substates as stochastic subdivisions of regular and persistent brain states. This allows for recurrent substates to be detected and characterized in terms of probability of occurrence and alternation profiles.

This allows us to address the fundamental question in neuroscience of how the brain transitions come about between different states, e.g., from wakefulness to deep sleep and anesthesia or to disease states such as coma and neuropsychiatric disorders (11–13). Solving this problem requires 2 things: 1) a deeper understanding and quantitative definition of what constitutes a brain state, e.g., the proposed characterization of the PMS space; and 2) what drives the transitions between brain states. This would allow for the possibility of forcing a transition using for example external stimulation like deep brain stimulation (DBS), multifocal transcranial direct current stimulation (tCDS) (14), or transmagnetic stimulation (TMS) (15–19).

In order to do this, whole-brain models are needed that can link the underlying anatomical connectivity with the functional dynamics obtained from neuroimaging (8, 9, 20–24). We show here that a generative whole-brain model can actually accurately fit the PMS space of the empirical data corresponding to different brain states. Specifically, we used a unique dataset of continuous neuroimaging data from healthy participants falling asleep during simultaneous functional magnetic resonance imaging (fMRI) and electroencephalography (EEG) (25). Using this dataset, we have recently used a Markovian data-driven analysis to discover the dynamic choreography between different whole-brain networks across the wake–non-REM sleep cycle (26). However, here we provide evidence that we can externally force transitions between different brain states by means of in silico stimulation of the whole-brain model. Last, as proof-of-concept, we use this method to demonstrate that it can find accurate ways to promote transition from one brain state to another (as characterized by fMRI), and in particular find ways to “awaken” the brain from deep sleep to wakefulness and vice versa.

## Results

In order to find accurate ways to promote a stimulus-driven transition from one brain state to another, we first provide a quantitative characterization of the dynamics underlying brain states, here called the PMS space, and then fit a whole-brain model to this. Finally, we exhaustively probe this model off-line in silico, aiming to find optimal stimulation strategies for forcing the transition from one specific brain state to another. What follows offers a synopsis of this methodology (described in detail in *Materials and Methods*).

**Fig. 1.**
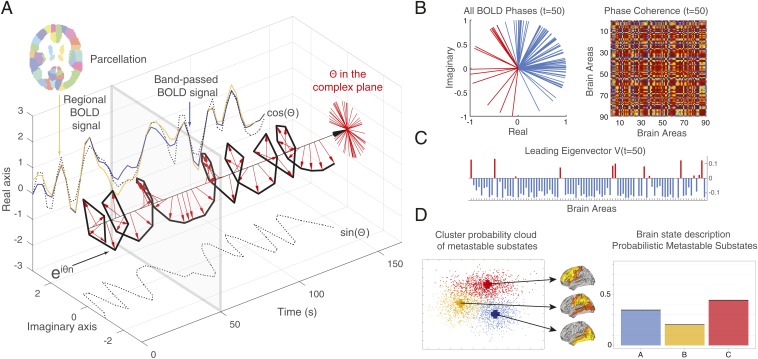
Computing the probabilistic metastable substate (PMS) space for whole-brain activity. Briefly, we use the leading eigenvector dynamics analysis (LEiDA) method, (*A*) where for every time point, *t*, in every brain region of each participant we extract the BOLD signal, compute the phase of the BOLD signal, and (*B*) compute the BOLD phase coherence matrix, dFC(*t*), between brain regions, and (*C*) extract the leading eigenvector V1(*t*) of this matrix. (*D*) To obtain a probabilistic metastable substate (PMS) space, we then take all of the leading eigenvectors for all time points in all participants and use an algorithm to cluster them (here, we show 3 clusters). More specifically, in *A*, we show for each of the 90 AAL parcels (shown in different colors), we extract the BOLD signal for a given region (here in orange). For each brain area, the original BOLD signal (orange) is first band-pass filtered between 0.02 and 0.1 Hz (blue) and then transformed into an analytic signal, which can be represented by its time-varying amplitude *A* and its phase θ (with real and imaginary components) using the Hilbert transform. The Hilbert phase, θ, can be represented over time in the complex plane by e^iθ^, where the real part is given by cos(θ), while the imaginary part by sin(θ) (black dotted lines). The red arrows represent the BOLD phase at each TR. As can be seen, much of the original BOLD signal is captured by the BOLD phase, cos(θ) (dotted line). (*B*) For a given time point (gray box shown at *t* = 50 in *A*), the *Left* panel shows the BOLD phases in all 90 AAL regions represented in the complex plane (i.e., the unit circle with real and imaginary axis, where all phases are centered at the same origin). The *Right* panel shows the phase coherence matrix at a given time *t* with the BOLD phase coherence (PC) between each pair of brain regions. (*C*) The leading (i.e., largest magnitude) eigenvector of this phase coherence matrix at time *t*, V1(*t*), is the vector that best captures the main orientation of all BOLD phases, where each element in V1(*t*) corresponds to the projection of the BOLD phase in each region into V1(*t*). The elements of V1(*t*) are colored according to their sign (red, positive; blue, negative; same color scheme in all phase representations). (*D*) Finally, to determine the PMS space, we gather all leading eigenvectors V1(*t*) for all participants across time as a low-resolution representation of the BOLD phase coherence patterns. We apply a clustering algorithm (*k*-means) to divide the sample into a predefined number of clusters *k* (here, *k* = 3). Each cluster is represented by a central vector (blue, orange, and red), which we take to represent a recurrent pattern of phase coherence, or substate, which occurs with a given probability. Any brain state can thus be represented by this PMS space.

The first step of our analysis has been to identify the PMS space, by using the leading eigenvector dynamics analysis (LEiDA) method (27), described in detail in *Materials and Methods* and in Fig. 1. Standard (FSL) tools were used to first extract, preprocess and average the blood oxygen level-dependent (BOLD) signals of 90 regions of interest (ROIs) defined in the Automated Anatomical Labeling (AAL) atlas. They included cortical and subcortical noncerebellar ROIs (nodes) that were considered for the estimation of dynamic connectivity. The average BOLD time series of each ROI were Hilbert-transformed to yield the phase evolution of the regional signals. The phase coherence for each pair of nodes at any given time can be defined as the cosine of the phase differences, the value of which varies from 1 to −1, for signals changing in exactly the same or opposite direction, respectively. This process yields a 3D matrix of NxNxT-size, where N = 90 and T indicates the number of image volumes acquired in all considered sessions. Clustering the large number of NxN dynamic functional connectivity (dFC) matrices may be in principle used for estimating metastable states. However, reducing the very large dimensionality of the coherence matrices significantly improves the signal-to-noise ratio and the reliability of any clustering or classification process aiming at the description of states. We have therefore used the aforementioned LEiDA method that relies on the leading eigenvector of each NxN coherence matrix.

The method relies on the extraction of the first (Nx1) eigenvector, V1, of each dFC matrix, from which one can reliably detect a discrete number of reduced dFC patterns by applying clustering across time points and subjects. Due to the symmetry of the dFC matrices, each leading eigenvector may be used in turn to estimate the corresponding dFC matrix. The obtained *k*-cluster centroids define the “metastable substates,” for which one can compute the probability of the centroid, as well as its transition probabilities.

We use this method in the cases of 2 different naturally occurring brain states: awake and deep sleep conditions obtained in healthy human participants measured with fMRI and EEG (25). The clustering as determined by the silhouettes criterion showed that 3 metastable substates were optimal for fitting both brain states. Nevertheless, it is entirely possible, albeit computationally very expensive, to fit the whole-brain model to a higher number of metastable substates, potentially revealing more of the known networks involved in sleep shown, e.g., in our previous Markovian data-driven results (26). Here, we used 3 metastable substates as a proof-of-concept.

The strategy of brain state definition and potentially of inducing a brain state transition is summarized in Fig. 1, demonstrating a probabilistic state space that may be underlying a given brain state, e.g., wakefulness, sleep, anesthesia, or disease.

**Fig. 2.**
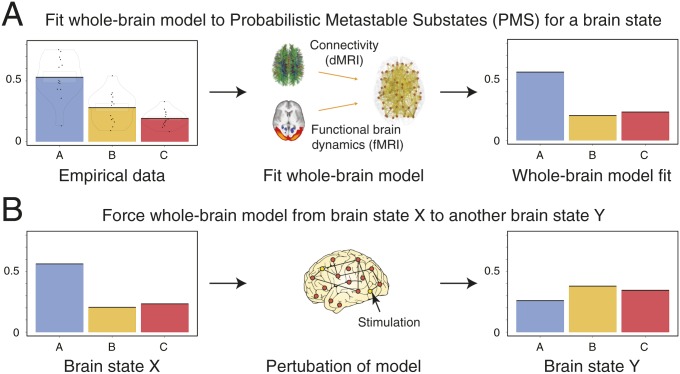
Schematic of how to model a brain state and force a transition between different brain states. This process involves 3 steps. (*A*) First, we empirically characterize a given brain state in terms of its probabilistic metastable substates (PMS) space (using the method shown in Fig. 1). This gives rise to 3 different substates (blue, orange, and red), which have different probabilities associated with each substate. Second, we fit a whole-brain model directly to this PMS space (*Materials and Methods*). (*B*) Third, the whole-brain model in source brain state X can be perturbed and stimulated exhaustively in order to promote and force a specific transition to a target brain state Y.

In order to promote or force transitions between brain states, we propose a framework to first characterize brain states (Fig. 1), then use whole-brain models to generate such brain states (Fig. 2*A*), and ultimately attempt to force a transition between them (Fig. 2*B*). Each point in the proposed framework represents an instantaneous snapshot of the whole brain at a point in time. The cloud, in turn, describes a global brain state, such as that during wakefulness or deep sleep. In Fig. 1*D*, the probabilistic points are projected onto a 2D space for visualization, but the original state space is of course likely to be higher dimensional.

By clustering the probabilistic points shown in Fig. 1*D*, we can generate substates from groups of points and express their probability (leftmost panel of Fig. 1*D*). Since such groups will still be changing over time, we call them PMS. In Fig. 2*A*, we show how we can fit a whole-brain model to this PMS space, which provides a probabilistic description of the spatiotemporal dynamics of the underlying functional fMRI time series. The whole-brain model links the structural anatomy [given by the diffusion MRI (dMRI) data] with the functional dynamics (given by the fMRI data) by adapting the free parameters (the effective conductivity of the anatomical fibers) to provide the optimal fit between the simulated and empirical PMS spaces [by using an appropriate probabilistic distance measure, namely symmetrized Kullback–Leibler (KL) distance].

In Fig. 2*B*, we show how the whole-brain model can be perturbed and stimulated exhaustively in order to promote and force a specific transition between 2 different brain states. Specifically, in what follows, we discuss the strategy of fitting the whole-brain model to the PMS space, and inducing a transition from one specific brain state to another.

### Optimal Spatiotemporal Fit of Whole-Brain Model to PMS Space.

The whole-brain, large-scale model consisted of local nodes representing local brain regions in a parcellation (*Materials and Methods*). The connectivity of different brain regions was constrained by the underlying anatomical connectivity matrix, i.e., the structural connectivity (SC), between those nodes. The SC matrix was obtained using diffusion MRI and tractography techniques (*Materials and Methods*), while the dynamics of each local brain area network were described by the normal form of a supercritical Hopf bifurcation, also known as the Landau–Stuart oscillator (28). The internal parameters of the whole-brain model (e.g., the coupling strength between nodes) can be optimized to fit the PMS space.

Indeed, the strategy here is to use the centroids of the empirical clusters defining the metastable substates, and to compute the simulated probabilistic measurements based on those empirical centers. In this way, we assure that the probabilistic measurements correspond exactly to the empirical metastable substates.

**Fig. 3.**
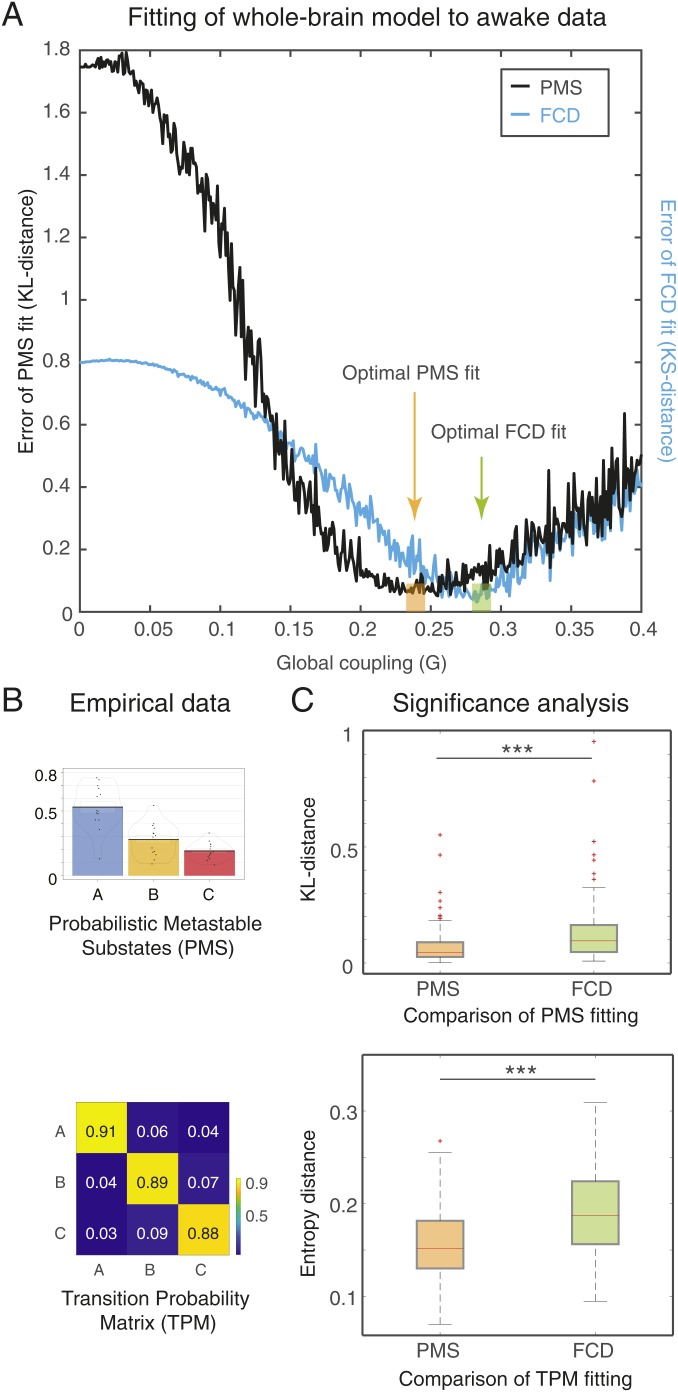
Results of fitting whole-brain model to probabilistic state space of resting-state wakefulness fMRI data. (*A*) The fit is determined when varying the single global coupling parameter, *G*, which scales the density of fibers expressed in the SC and can be interpreted as the synaptic conductivity of each single fiber. Here, we show 2 measurements of fitting as a function of *G*: 1) the phase-coherence–based functional connectivity dynamics (FCD), and 2) the probabilistic metastable substates (PMS) space. The best fit of the FCD can be found by computing the Kolmogorov–Smirnov (KS) distance (black line), while the fit to PMS space can be computed using the symmetrized Kullback–Leibler (KL) distance between the empirical probabilities and the simulated probabilities generated by the model using the same empirical centroids (blue line). The optimal fit of for PMS is with *G* = 0.245. (*B*) We show the PMS space and the TPM when using 3 substates. (*C*) We found that the PMS fitting of the empirical data are significantly better than the FCD fitting (upper figure). The transition probabilities are also significantly better fitted as measured with Markov entropy distance (lower figure). Please note how the values of the diagonal of the transition matrix (i.e., the probability of remaining in the same state) are much higher than the probabilities of switching states, reflecting the metastable nature of substates.

Fig. 3*A* shows the performance of the whole-brain model for fitting data of wakefulness state. In this case, we exhaustively explored the parameter *G*, which scales the interarea coupling, thereby determining the dynamical working point of the system. The *G* coupling parameter scales the density of fibers expressed in the SC and can be interpreted as an indicator of axonal conductivity, under the simplistic assumption that conductivities of axons related to long-range connectivity are equal across the brain, assuming similar myelination density.

Fig. 3*A* shows 2 measurements of fitting as a function of *G*: 1) the phase-coherence–based functional connectivity dynamics (FCD); and 2) the PMS. The first is computed by collecting the upper triangular elements of the time-resolved phase coherence connectivity dFC(*t*) matrices (over all participants) and then use the Kolmogorov–Smirnov (KS) distance to compare these empirical distributions with the corresponding simulated distributions from the model. The minimum value of this KS distance corresponds to the optimal fitting of the spatiotemporal characteristics and is obtained at *G* = 0.283. In this case, where the whole-brain model was fitted to the FCD, the optimal minimum turned out not to be the best fitting of the spatiotemporal dynamics as characterized by PMS.

Instead, in the second case, we obtained more accurate results by extracting the empirical PMS, shown in Fig. 3*B* with the PMS space as well as the transition probability matrix (TPM). To find the optimal minimum for the PMS, we computed the symmetrized KL distance between the empirical probabilities and the simulated probabilities, generated by the model from the same empirical centroids. This was a stringent criterion to ensure that we would get the best possible fit. Indeed, the optimal PMS fitting of the empirical data were obtained at the minimum of *G* = 0.245, which clearly was a significantly better fit than that of the FCD (Fig. 3 *C*, *Upper*). Furthermore, the transition probabilities are also significantly better fitted as measured with the Markov entropy distance (Fig. 3 *C*, *Lower*). Note that values of the diagonal of the transition matrix (i.e., the probability of remaining in the same state) are much higher than the probabilities of switching states. This is important as it reflects the metastable character of the substates.

Overall, these results showed that the PMS-based measurements for characterizing a brain state provide the most significantly accurate working point of the whole-brain model to account for the most detailed spatiotemporal dynamical characteristics of the brain activity defining a brain state.

### Optimizing the Whole-Brain Model by Using Effective Connectivity.

The third step in modeling the spatiotemporal PMS space is to derive 2 different optimized whole-brain models for 2 radically different brain states. Here, as a proof-of-concept, we used 2 naturally occurring significantly different states, namely, wakefulness and deep sleep (25).

**Fig. 4.**
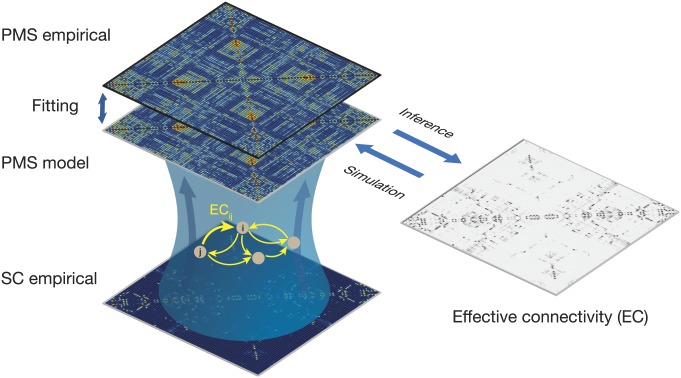
Fitting whole-brain using effective connectivity (EC). We improved on the fitting procedure by optimizing the effectiveness of the synaptic connections between brain regions as specified by the SC. We compute the distance between the model and the empirical grand average phase coherence matrices, and adjust each structural connection separately using a gradient-descent approach (see *Materials and Methods* for details). The model is run repeatedly with the updated EC until convergence.

Fig. 4 demonstrates the improvements in the aforementioned fitting procedure, by optimizing the effectiveness of the synaptic connections between brain regions [effective connectivity (EC)]. Specifically, we computed the distance between the model and the empirical grand average phase coherence matrices, and adjust each structural connection separately using a gradient-descent approach (see *Materials and Methods* for details). The model was run repeatedly with recursive updates of EC until convergence was reached (*Materials and Methods*).

**Fig. 5.**
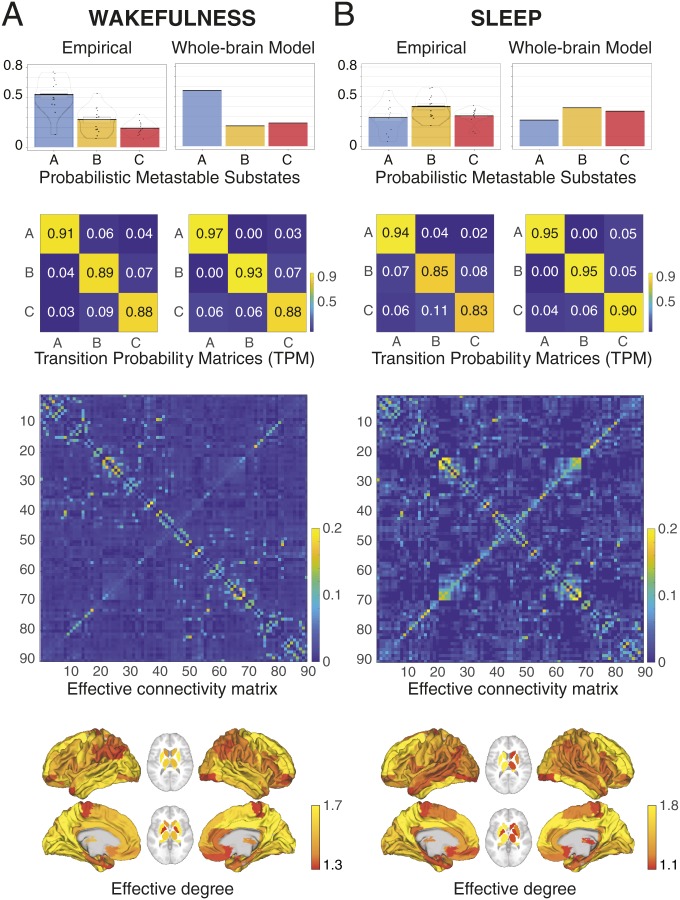
Fitting a whole-brain model to 2 radically different brain states (wakefulness and sleep) measured using fMRI. The figure shows the results obtained for wakefulness (left column; *A*) and sleep (right column; *B*) comparing the empirical results with the output of the fitted whole-brain EC model. In each row, we show the PMS space, transition probability matrices, EC matrix, and the effective degree. As can be seen, there is an excellent fit between model and empirical data for both brain states (e.g., compare the bar plots for the PMS spaces). Please also note how different the EC matrices are for the 2 states (bottom 2 rows).

Fig. 5 shows the results obtained during wakefulness (Fig. 5*A*) and sleep (Fig. 5*B*), with the first row showing the PMS space and the second row, the TPM, and with left column showing the empirical results and the right column, the fit of the whole-brain model. Below this, we show EC matrix and the effective degree. For the whole-brain model fit of PMS space, we found a KL distance of 0.0169 for wakefulness, and 0.0045 for sleep. Similarly, for whole-brain fit of the TPM, we found a Markov entropy distance of 0.098 for wakefulness and 0.109 for sleep. Also note how different the EC matrices are for the 2 states (bottom 2 rows).

### Awakening: Forcing a Brain State Transition.

The previous results show that we can successfully create 2 whole-brain models with excellent fit to the empirical fMRI data from 2 radically different brain states. However, the most important finding of this study is the demonstration of the possibility to force a transition from one whole-brain model to another via external stimulation.

**Fig. 6.**
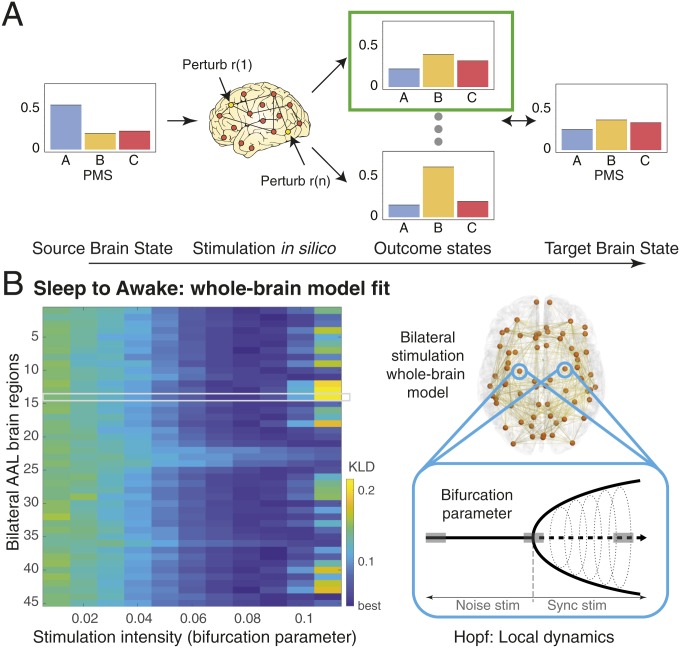
Schematic of strategy for forcing transition between source and target brain states. (*A*) The brain regions in the whole-brain model of the source state can be systematically stimulated, and the results can be compared to the target state. Specifically, in the local region Hopf model, it is easy to perturb the model by simply changing the bifurcation parameter (29). (*B*) The stimulation intensity, i.e., the strength of the perturbation, is directly related to the amount of shifting the local bifurcation parameter (*Right*; *Materials and Methods* and ref. 30). The composite results are shown *Left* of stimulating the whole-brain EC model bilaterally. We show the KL distance obtained for brain state transition fitting when perturbing separately each of the 45 regions (using bilateral stimulation) with different stimulation intensities in source state (deep sleep). Here, we have highlighted 1 region in a gray region, which is being stimulated while the other regions are kept at their normal bifurcation parameter. The color scale for the results shows the level of fitting with the target state (wakefulness), i.e., lower values (deep blue) correspond to the most effective transitions.

Fig. 6 summarizes the process of forcing a transition between 2 brain states (source and target). We systematically perturbed the brain regions in the whole-brain model of the source state and compared the resulting PMS space for this model with the empirical data for the other target state (Fig. 6*A*). Specifically, the Hopf model allowed an effective way of perturbing the model by simply changing the bifurcation parameter in a given brain region (29). The stimulation intensity, i.e., the strength of the perturbation, is directly related to the amount of shifting the local bifurcation parameter (Fig. 6 *B, Right*, *Materials and Methods*, and ref. 30). We perturbed the model bilaterally in Fig. 6 *B*, *Left*, which shows the levels of brain state transition fitting for perturbing separately each of the 45 regions (since it is bilateral stimulation) with different stimulation intensities in source state (deep sleep). The color scale of Fig. 6 *B*, *Left*, shows the level of fitting with the target state (wakefulness), i.e., lower values (blue) correspond to an effective transition.

**Fig. 7.**
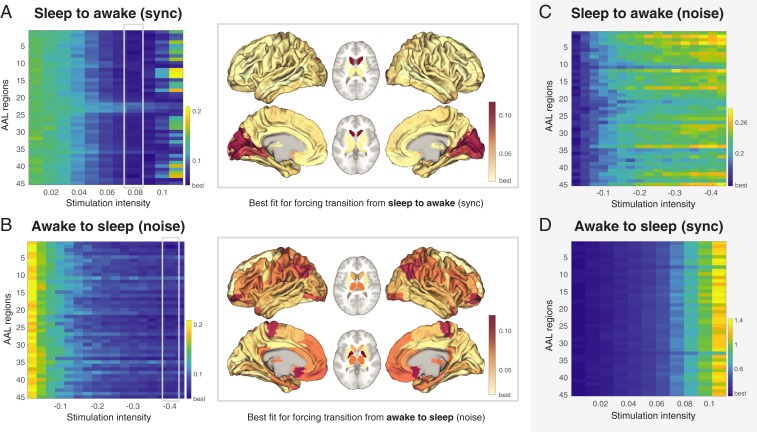
Main results of forcing a transition between brain states. We used 2 different protocols for external stimulation, synchronization (sync) and noise, which shifted the local bifurcation parameter to positive and negative values, respectively. (*A*) Shown are the results of forcing a transition from source state (deep sleep) to target state (wakefulness) using a synchronization protocol where positive values of the local bifurcation parameter force local oscillations that promote the possibility of more synchronization across the whole brain. The color scale indicates the KL distance between source and target state with lower values indicating a better fit (more blue). As can be seen, a transition from deep sleep to wakefulness is promoted when perturbing most brain regions with sufficient stimulation intensity (a = 0.08). At *Right*, we show the ability of brain regions to promote transition at this stimulation intensity. It is clear from the results that while many regions are able to promote a transition (given sufficient stimulation), other regions are less suitable for this (see burgundy areas). (*B*) Shown are the results of forcing the opposite transition from source state (wakefulness) to target state (deep sleep) using a noise protocol, where negative values of the local bifurcation parameter force local oscillations that promote the possibility of more noise and less synchronization across the whole brain. The results show more specificity for making the wakeful brain move to deep sleep than for the inverse, with the *Right* showing the ability of brain regions to promote transition at the stimulation intensity of a = −0.4 (note the increase in burgundy areas). Please also note how the noisy protocol needs larger absolute values than the synchronization protocol. (*C*) In contrast, transitions are not always possible, such as when using the opposite protocols for forcing transitions. In particular, when using the noise protocol to force a transition from deep sleep to wakefulness increases in stimulation intensity lead to higher KL distances, i.e., poorer fit, indicated by an increase in the colors to more yellow from blue. Also note that the color scale is different between *A* and *C*, with the first column in each figure having the identical numerical KL distances (corresponding to the nonperturbation case) but appearing in different colors due to different color scales. (*D*) Similarly, it is not possible to force a transition from wakefulness to deep sleep when using the synchronization protocol, as shown by the monotonic increase in KL distance.

For the main transition results, we used 2 different protocols for external stimulation, synchronization and noise, which shifted the local bifurcation parameter to positive and negative values, respectively. Fig. 7*A* shows the results for forcing a transition from source state (deep sleep) to target state (wakefulness) using a synchronization protocol where positive values of the local bifurcation parameter force local oscillations that promote the possibility of more synchronization across the whole brain. The color scale indicates the KL distance between source and target state with lower values indicating a better fit. This promotes a transition from deep sleep to wakefulness when perturbing most brain regions with sufficient stimulation intensity (a = 0.08). In 7 *A*, *Right*, we show a rendering of brain regions to promote transition at this stimulation intensity. It is clear from the figures that while many regions are able to promote a transition (given sufficient stimulation), other regions are less suitable for this (see burgundy areas). Importantly, in Fig. 7*C*, we show that using the noise protocol to force a transition from deep sleep to wakefulness is not possible, with an increase in stimulation intensity leading to higher KL distances, i.e., poorer fit, indicated by an increase in the colors to more yellow from blue. Also note that the color scale is different between Fig. 7 *A* and *B* compared to Fig. 7 *C* and *D*, with the first column in each figure having the identical numerical KL distances (corresponding to the nonperturbation case) but appearing in different colors due to different color scales.

Fig. 7*B* shows the results for forcing a transition from source state (wakefulness) to target state (deep sleep) using a noise protocol, where negative values of the local bifurcation parameter force local oscillations that promote the possibility of more noise and less synchronization across the whole brain. The results show more specificity for making the wakeful brain move to deep sleep than for the inverse, with the right panel showing the ability of brain regions to promote transition at the stimulation intensity of a = −0.4 (note the increase in burgundy areas). Importantly, Fig. 7*D* shows that using the synchronization protocol does not in any case result in a transition from wakefulness to deep sleep (note the increase in KL distance). This could be interpreted that it is probably much easier, and thus unspecific, to promote a transition from sleep to awake than vice versa.

**Fig. 8.**
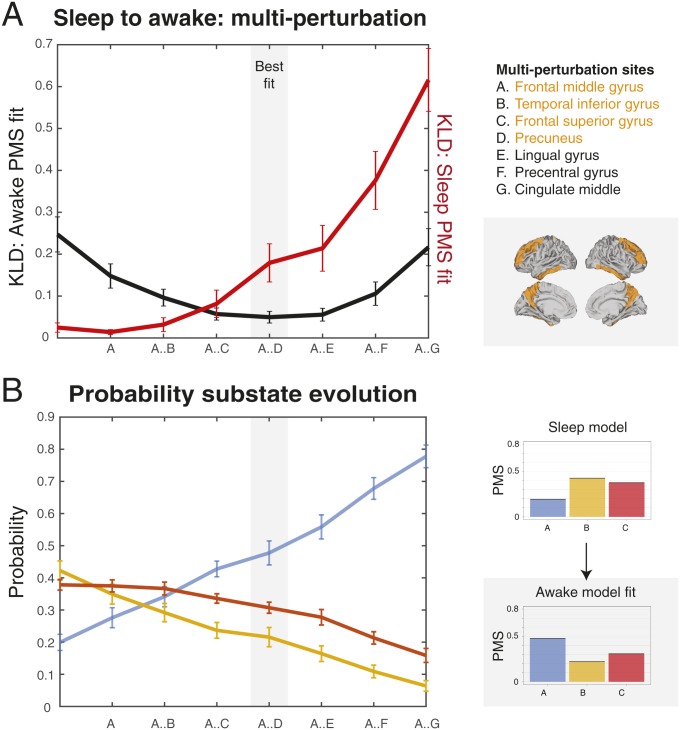
Stimulation of multiple regions with weaker stimulation intensity can produce equal or better results to single-site stimulation. We used a multisite stimulation protocol with a greedy strategy to find the best combination of multiple brain regions to force a transition between states (deep sleep to wakefulness). Using the synchronization protocol but at the weaker stimulation intensity of a = 0.02, we identified the region that best fit the target PMS space and let this region continue to be stimulated while we looked for the best region among the rest in this new condition. The process was iterated over 7 steps. (*A*) The figure shows how the combination of multisite stimulation reaches its best fit using 4 bilateral stimulated regions (in gray shaded area) and then starts to get worse when more regions are added. The best fit is comparable to the best fit for single-site stimulation at higher stimulation intensity (Fig. 7*A*). This is shown by the black line indicating the level of fit to the target PMS space (wakefulness) and reaches a minimum for 4 regions (listed in orange and rendered on the brain in orange). In contrast, the red line indicates the level of fit to the source PMS space (deep sleep), which gets monotonically worse with more stimulation sites. (*B*) To better understand how the multisite stimulation promotes transition, the figure plots the evolution of the 3 substates (blue, orange, and red) in the source (deep sleep) and target (wakefulness) states as a function of multiregion stimulation. The black line represents the probability of state A, which increases with number of regions stimulated, while orange and red lines represent the decreasing probabilities of substates B and C. This nicely fits the transition between the probability of the PMS in source and target states, shown on the *Right*, with the optimal fit found for the same 4 regions.

Finally, we explored whether stimulating multiple regions with weaker stimulation intensity would produce equal or better results. Fig. 8 shows the results of using this multisite stimulation protocol using a greedy strategy (31) to find the best combination of multiple brain regions for forcing a transition between states (deep sleep to wakefulness). Using the synchronization protocol but at the weaker stimulation intensity of a = 0.02, we identify the region that best fit the target PMS space and let this region continue to be stimulated while we look for the best region among the rest in this new condition. The process was iterated over 7 steps. As shown in Fig. 8*A*, the combination of multisite stimulation reaches its best fit (comparable to the best fit for single-site stimulation at higher stimulation intensity; Fig. 7*A*) using 4 bilateral stimulated regions (frontal middle gyrus, temporal inferior gyrus, frontal superior gyrus, and precuneus, shown rendered in the right upper panel) and then starts to get worse when more regions are added. This is shown by the black line, which indicates the level of fit to the target PMS space (wakefulness) and reaches a minimum for 4 regions. On the other hand, the red line indicates the level of fit to the source PMS space (deep sleep) and gets monotonically worse with more stimulation sites. Finally, in order to better understand how the multisite stimulation promote transition, in Fig. 8*B*, we plot the evolution of the 3 substates in the source (deep sleep) and target (wakefulness) states as a function multiregional stimulation. The blue line represents the probability of substate A, which increases with number of regions stimulated, while orange and red lines representing probability of substates B and C decrease. This nicely fits the transition between the lifetimes of the PMS in source and target states, shown on the *Right*, with the optimal balance for lifetimes found with 4 stimulated regions.

## Discussion

We successfully used a whole-brain model to fit the PMS space of the 2 radically different brain states of sleep and wakefulness. The states were extracted from a unique continuous neuroimaging data of healthy participants falling asleep during simultaneous fMRI and EEG (25). We provided the evidence that in silico stimulation of this whole-brain model can be used to show where to force transitions between different brain states and thus “awaken” the brain from deep sleep to wakefulness and vice versa.

We provided evidence primarily for role of single regions to promote this transition but also provided proof-of-principle that multisite stimulation can achieve similar results with less stimulation in each site. As expected, we found an optimum number of regions that will promote a transition between states, while adding more regions will significantly worsen the transition probability, reflecting the metastable nature of the brain states. Delivering multisite stimulation to the brain is by its very nature more difficult with techniques such as DBS but perhaps possible with a multifocal tDCS array (14). Still, at this point it is too early to speculate whether single or multisite stimulation would be more optimal. In ongoing work, we have also demonstrated that we can achieve awakening with more biophysical realistic models such as the dynamic mean field model, which speaks to the potential for clinical benefit.

Interestingly, the results show that it is easier to awaken the model, i.e., promote a transition from sleep to wakefulness, rather than promote a transition in the opposite direction. It requires less stimulation in more regions. Evolutionarily, this makes sense given that in order to promote survival we have to be able to easily wake from slumber, while falling asleep easily could be seen as less critical.

More broadly, these results have to be seen in the context of the well-known Latin statement “post hoc, ergo propter hoc” (“after this, therefore because of this”), which is a classical logical fallacy, confusing correlation and temporal sequences with causality. There have long been many spectacular examples of cases, in which the effect may even precede its cause, as in the case of backward causation (e.g., the retrocausality in the fields of quantum mechanics). Richard Feynman (F)—with his F-clocks and F-gates—was one of the historical physicists who attempted to understand so-called plateaus of complexity (POCs), i.e., the physical states of complex systems, by enforcing state transitions and describing the POCs associated with them (32). One thing became clear over time: state transitions in any discipline can only be correctly interpreted, predicted, or even successfully enforced, if the states themselves are well understood at a necessary complexity level, and the self-organization and dynamics of the systems can be modeled, potentially leading to theories predicting empirical data.

Still, it goes without saying that understanding states and their dynamics in brain-like systems makes physics to appear as an “easy” matter (32). The organization of the brain daily challenges most of our efforts to study it, as its understanding requires concurrent studies of local and global networks at different spatiotemporal scales, as well as of their dynamical evolution over time. At the macroscopic level, the developments of neuroimaging over the last few decades has become a valuable source of empirical data (e.g., ref. 33), but quantitative description of such data, modeling of systems, and even perturbing them in silico for understanding causal relationship between neural and cognitive states is a field in its early childhood, albeit with promising perspectives (23, 24, 31, 34, 35).

State definition in systems neuroscience justifiably has drawn and continues to draw a great deal of attention. Attempts were made to define a brain state in many different ways, often by using heuristic definitions of signals or signal combinations, and points in a empirically defined state space characterizing the activity of the brain at a given time (2–5), by defining attractors of interacting brain regions (5, 6). Other approaches utilized the whole-brain connectomic measurements including dFC (36–39). However, most of these approaches fail to capture and exploit the intriguing role of various band-limited–power neurophysiological signals and the spatiotemporal richness of neuroimaging data.

The PMS space strategy we describe here captures an unusually large proportion of data as an ensemble or probabilistic “cloud” in a state space, which can be decomposed into so-called metastable substates (27, 40), which are temporarily stable but variable as a function of time (30, 41, 42). Fitting was optimized by fine-tuning the model on the basis of the EC.

A clear advantage of using such data-constrained whole-brain model is its potential use for studying stimulation-induced state transitions, as it enables an exhaustive search and optimization of all underlying parameters and locations in silico, and it may ultimately offer insights into the self-organization of widespread networks. Such insights may be impossible to gain even with direct electrical stimulation (DES) of brain sites in experimental animals, or occasionally in human patients, as DES has been shown to violate the most basic principles of cortical microcircuits, disrupting cortico-cortical signal propagation by silencing the output of any neocortical area whose afferents are electrically stimulated (43–45). Stimulation of lateral geniculate nucleus (LGN), for example, will yield fMRI maps with positive BOLD responses, in the striate cortex, colliculus, and pulvinar, and negative bold responses in all extrastriate areas, disynaptically connected to LGN. Intracranial recordings, concurrently carried out with fMRI, consistently confirm that a short excitatory response occurring immediately after a stimulation pulse was followed by a long-lasting spiking inhibition. This inhibition is synaptic, rather than due to excitability changes, as injection of GABA antagonists, such as bicuculine, restores the stimulation-induced excitatory responses.

It has long been known that, in the cortical canonical microcircuits, excitation and inhibition are inseparable. Sensory stimulation will first excite glutamatergic and, ∼2 ms later, GABAergic neurons (46). Both have a strong recurrent self-amplification, and the output of the microcircuit depends on so-called excitation–inhibition (E–I) balance at any given time. Electrical stimulation of the cortical afferents, nulls the excitation differences between E- and I-neurons, activating both groups simultaneously, and shunting the output of the microcircuits through the aforementioned powerful synaptic inhibition and before the initiation of the recurrent self-amplification (44, 47). Not surprisingly thus, the stimulation of the LGN in monkeys in DES-fMRI experiments demarcated all monosynaptic targets of the stimulated brain site, or alternatively a selection of polysynaptic nuclei reached through the antidromic stimulation of collaterals of cortical infragranular projection neurons. It follows that DES in animals or humans propagates through cortico-subcortico-cortical pathways, rather than cortico-cortical connectivity. The often unpredictable active connectivity combined with the very fact that we can still not precisely define the element activated by DES (19, 48). This explains the fact that a large amount of Parkinson patients suffer from adverse stimulation effects (49). Understanding states and optimizing in silico stimulations can only be instructive for future attempts to deal with the DES technology and interpretation of its results.

The approach presented here may open new, exciting possibilities for discovering new stimulation targets for DBS and TMS in disease. A stimulation strategy that minimizes the unavoidable aforementioned DES effects by maximizing the network nodes that can be activated by direct monosynaptic or indirect cortico-subcortico-cortical paths may indeed provide substantial alleviation of patient suffering. Moreover, the approach could be used as a principled way to rebalance human brain activity in health and disease (15) by causally changing the cloud of metastable substates from that found in disease to the health in order to promote a profound reconfiguration of the dynamical landscape necessary for recovery, rather than having to identify the original point of insult and its repair.

In addition, the presented approach significantly broadens the findings from previous whole-brain models of simultaneous neuroimaging activity and direct ON–OFF DBS alleviating symptoms of brain disease that have revealed trigger points and underlying brain networks (50–52). Deco et al. (29, 31), for instance, have lesioned and perturbed brain regions and networks in whole-brain models in silico, demonstrating the causal contribution of brain regions to the ability of the human brain to efficiently integrate information over time.

Using the proposed framework, further awakenings can be imagined, perhaps even promoting the “awakening” of locked-in patients. A whole host of stimulation technologies such as DBS and TMS could potentially be used to force such transitions between health and disease. However, it might also be possible to use targeted neurotransmission to the same effect, now that whole-brain models have successfully included neurotransmission (24).

Overall, the methods and results presented here may eventually allow us to build causative whole-brain models that can characterize all brain states, including levels of consciousness, disease, and cognitive states. In particular, this could have \great clinical utility, given that it could provide a principled way of discovering how to force a transition between 2 brain states.

## Materials and Methods

### Experimental Data.

We based our research on BOLD fMRI data recorded in Frankfurt (Germany) where participants fell asleep during a simultaneous EEG-fMRI scanning session. The experimental results were already described in detail in previous publications (25). All subjects gave written informed consent with approval by the local ethics committee. For the present study, we only considered the subset of subjects who reached deep sleep (stage N3). A summary of the technical and experimental aspects of data acquisition and preprocessing can be found in *SI Appendix*.

### LEiDA.

First, we calculated a phase coherence matrix at each time point to capture the amount of interregional BOLD signal synchrony at any given time point, for all subjects and conditions (awake and N3 deep sleep). Specifically, the BOLD time series of each ROI, corresponding to well-demarcated brain areas and structures, were high-pass filtered and subsequently Hilbert-transformed to yield the phase evolution of the BOLD time course of each node (i.e., ROI). The phase coherence dFC(*n*,*p*,*t*) between each *n* and *p* pair of nodes at time *t* was then estimated by calculating the cosine of the phase difference, as shown in Eq. **1**:

dFC(n,p,t)=cos(θ(n,t)−θ(p,t)). [1]

Because Hilbert transform expresses any given signal *x* in polar coordinates, i.e., *x*(*t*) = *A*(*t*) × cos(θ(*t*)), using the cosine function, 2 nodes *n* and *p* with temporarily aligned BOLD signals (i.e., with similar angles) at a given repetition time (TR) will have a phase coherence value close to 1 [cos(0°) = 1], while nodes with orthogonally developing BOLD signals (e.g., one increasing at 45° and the other decreasing at 45°) will have zero phase coherence [i.e., cos(90°) = 0]. The resulting dFC(*t*) for each subject in each condition is a thus 3D matrix with size NxNxT, where N = 90 is the number of demarcated brain areas, and T is the total number of time points (different for each subject and each condition). Notably, the phase coherence matrix dFC(*t*) at each time point is undirected and, as such, symmetric across the diagonal. The latter property permits strong reduction of the dimensionality of coherence matrix by calculating its leading eigenvector, V1(*t*), at each time point (53–55). The Nx1 leading eigenvector captures the instantaneous dominant connectivity pattern of the dFC(*t*), as can be easily observed by calculating its outer product V1.V1^T^. This strategy substantially reduces the dimensionality of the data, from NxN to Nx1, compared to the traditional approaches considering all values of the connectivity matrix (36, 39, 56). For further details about LEiDA, the reader is invited to consult the work of Cabral and colleagues (27, 55).

Upon computing the leading eigenvector of the phase coherence matrix dFC(*t*) for each TR, the next step in our analysis was to identify recurrent FC patterns that may reflect metastable substates. A discrete number of FC patterns was detected by clustering the leading eigenvectors V1(*t*) from the collapsed awake and deep-sleep data fMRI data (2 conditions) including all subjects. The *k*-means clustering algorithm was run with cluster-number *k* varying from 2 to 8 clusters. Clustering the leading eigenvectors yields *k Nx1* dimensional cluster centroids V_c_ the outer product, V_c_V_c_^T^, of which represents the dominant connectivity pattern in each cluster, with the V_c_ elements depicting the contribution of each node (brain ROI) to the community structure. To facilitate visualization and interpretation of substates, the cluster centroid vectors V_c_ were rendered onto a cortical surface using the Human Connectome Project (HCP) Workbench. For our current analysis, the optimal number of clusters according to various criteria, such as Silhouette, minimal *P* value for significant differences between condition probabilities were *k* = 3.

Following the identification of metastable substates, we computed the probability of occurrence of each FC state in each condition. The probability of occurrence (or fractional occupancy) is simply the ratio of the number of epochs assigned to a given cluster centroid V_c_ divided by the total number of epochs (TRs) in each experimental condition (which is the same in all experimental conditions). The probabilities were calculated for each subject, in each experimental condition and for the whole range of the explored clustering conditions.

In addition, we computed the switching matrix, which captures the trajectories of PMS dynamics in a directional manner. In more detail, it indicates the probability of, being in a given substate (rows), transitioning to any of the other substates (columns). Differences in probabilities of occurrence and probabilities of transition were statistically assessed between conditions using a permutation-based paired *t* test. This nonparametric test uses permutations of group labels to estimate the null distribution. The null distribution is computed independently for each experimental condition. For each of 1,000 permutations, a *t* test is applied to compare populations and the significance threshold α = 0.05 was used.

### Whole-Brain Computational Model.

We simulated the BOLD activity at the whole-brain level by using so-called Hopf computational model, which emulates the dynamics emerging from the mutual interactions between brain areas, considered to be interconnected on the basis of the established graphs of anatomical SC (30, 57). The model consists of 90 coupled dynamical units (ROIs or nodes) representing the 90 cortical and subcortical brain areas from the aforementioned AAL parcellation. The local dynamics of each brain area (node) is described by the normal form of a supercritical Hopf bifurcation, also called a Landau–Stuart oscillator, which is the canonical model for studying the transition from noisy to oscillatory dynamics (28). When coupled together using brain network architecture, the complex interactions between Hopf oscillators have been shown to successfully replicate features of brain dynamics observed in electrophysiology (58, 59), magnetoencephalography (60), and fMRI (30, 57).

The dynamics of an uncoupled node *n* is given by the following set of coupled dynamical equations, which describes the normal form of a supercritical Hopf bifurcation in Cartesian coordinates:

$$\frac{dx_{n}}{dt}=\left[a_{n}-x_{n}^{2}-y_{n}^{2}\right]x_{n}-\omega_{n}y_{n}+\beta \eta_{n}\left(t\right),$$ [2]

$$\frac{dy_{n}}{dt}=\left[a_{n}-x_{n}^{2}-y_{n}^{2}\right]y_{n}+\omega_{n}x_{n}+\beta \eta_{n}\left(t\right),$$ [3]

where $\eta_{n}\left(t\right)$ is additive Gaussian noise with SD β. This normal form has a supercritical bifurcation *a*_*n*_ = 0, so that if *a*_*n*_ > 0 the system engages in a stable limit cycle with frequency *f*_*n*_ *= ω*_*n*_/2π and when *a*_*n*_ < 0 the local dynamics are in a stable fixed point representing a low-activity noisy state. Within this model, the intrinsic frequency $\omega_{n}$ of each node is in the 0.04- to 0.07-Hz band (*n* = 1, …, 90). The intrinsic frequencies were estimated from the data, as given by the averaged peak frequency of the narrowband BOLD signals of each brain region.

To model the whole-brain dynamics, we added an additive coupling term representing the input received in node *n* from every other node *p*, which is weighted by the corresponding SC *C*_*np*_. This input was modeled using the common difference coupling, which approximates the simplest (linear) part of a general coupling function. Thus, the whole-brain dynamics was defined by the following set of coupled equations:

$$\frac{dx_{n}}{dt}=\left[a_{n}-x_{n}^{2}-y_{n}^{2}\right]x_{n}-\omega_{n}y_{n}+G\sum_{p=1}^{N}C_{np}\left(x_{p}-x_{n}\right)+\beta \eta_{n}\left(t\right),$$ [4]

$$\frac{dy_{n}}{dt}=\left[a_{n}-x_{n}^{2}-y_{n}^{2}\right]y_{n}+\omega_{n}x_{n}+G\sum_{p=1}^{N}C_{np}\left(y_{p}-y_{n}\right)+\beta \eta_{n}\left(t\right),$$ [5]

where *G* denotes the global coupling weight, scaling equally the total input received in each brain area. We fixed the noise SD to *β* = 0.02 and the mean SC to <*C*> = 0.2, in order to be in the same range of parameters previously explored in Deco et al. (30). While the oscillators are weakly coupled, the periodic orbit of the uncoupled oscillators is preserved. Notably, we do not address here the case of nonlinear coupling, in which the next nonvanishing higher-order term following a Taylor expansion of the full coupling should be considered (61, 62). The variable *x*_*n*_ emulates the BOLD signal of each node *n*. The global coupling parameter *G* is the control parameter with which we adjusted the model to the dynamical working region where the simulations optimally fit the empirical data (30, 63).

### Empirical Fitting.

#### Comparing empirical and simulated grand-averaged static functional connectivity.

The comparison was measured by computing the Pearson correlation coefficient between corresponding elements of the upper triangular part of the empirical and simulated grand-averaged functional connectivity (FC).

#### Comparing empirical and simulated FCD.

We measure KS distance between the upper triangular elements of the empirical and simulated FCD matrices (accumulated over all participants). For a single subject session where M time points were collected, the corresponding phase-coherence based FCD matrix is defined as a MxM symmetric matrix whose (t1, t2) entry is defined by the cosine similarity between the upper triangular parts of the 2 matrices dFC(t1) and dFC(t2) (previously defined; see above). For 2 vectors **p**_1_ and **p**_2_, the cosine similarity is given by (**p**_1_.**p**_2_)/(||**p**_1_||||**p**_2_||). Epochs of stable FC(*t*) configurations are reflected around the FCD diagonal in blocks of elevated inter-FC(*t*) correlations. The KS distance quantifies the maximal difference between the cumulative distribution functions of the 2 samples.

#### Comparing empirical and simulated probability metastable space state measurements.

For comparing the probabilities of the metastable states, i.e., the probabilities of the extracted empirical centers after clusterization, we used a symmetrized KL distance between the simulated and empirical corresponding probabilities, i.e.:

$$KL\left(P_{emp},P_{sim}\right)=0.5\left(\sum_{i}P_{emp}\left(i\right)\ln\left(\frac{P_{emp}\left(i\right)}{P_{sim}\left(i\right)}\right)+\sum_{i}P_{sim}\left(i\right)\ln\left(\frac{P_{sim}\left(i\right)}{P_{emp}\left(i\right)}\right)\right),$$ [6]

where *P*_*emp*_(*i*) and *P*_*sim*_(*i*) are the empirical and simulated probabilities on the same empirical extracted metastable substates *i*.

#### Comparing empirical and simulated transition probabilities between metastable substates.

We calculated the entropy rate S of a Markov chain, with N states and transition matrix P. The rate entropy S is given by the following:

$$S=S_{1}+S_{2}+\ldots +S_{N},$$ [7]

where

$$S_{i}=-p\left(i\right)\sum_{j=1}^{N}P\left(i,j\right)\text{log}P\left(i,j\right).$$ [8]

The probability p(i) represents the stationary probability of state i. For long realizations of the Markov chain, the probabilities of each state converge to the stationary distribution p, which is solution of the following equation:

$$P^{T}p=p.$$ [9]

Thus, the stationary distribution is the eigenvector of the transpose of the transition matrix with associated eigenvalue equal to 1. A Markov model that makes a lot of transitions has a large rate entropy, while a Markov model that barely transits has low entropy. For each transition matrix, we obtained the stationary distribution, and then calculated the entropy rate. The final measure comparing the 2 TPMs is just defined by the absolute value of the difference between both respective Markov entropy values.

#### Methods for updating EC.

We derived 2 whole-brain models, namely, 1 for accounting the awake condition and another for accounting the N3 deep sleep condition. In both cases, we optimized the EC between brain regions. Specifically, we compute the distance between the model $FC_{ij}^{phases\_mod}$and empirical $FC_{ij}^{phases\_emp}$ grand-averaged phase coherence matrices, and adjust each structural connection separately with a gradient-descent approach, thereby transforming structural into effective connections. The model is run repeatedly with the updated EC until the fit converges toward a stable value.

We start with the anatomical connectivity obtained with probabilistic tractography from dMRI and use the following procedure to update:

$$C_{ij}=C_{ij}+\varepsilon \left(FC_{ij}^{phases\_emp}-FC_{ij}^{phases\_mod}\right).$$ [10]

We update all connections in order to access the potential missing components of the anatomical connectivity obtained with dMRI, which is known to miss connections in the opposite hemisphere, given that most known tracts are bilateral. We use ε=0.01 and the grand average phase coherence matrix is defined as follows:

$$FC_{ij}=\langle \text{cos}\left(\varphi_{j}\left(t\right)-\varphi_{i}\left(t\right)\right)\rangle ,$$ [11]

where $\varphi_{j}\left(t\right)$ is the phase of the BOLD signal in brain region *j* at time *t* extracted with the Hilbert transform, with the bracket denoting the average across time.

## Supplementary Material

Supplementary File
