# Supplementary material for: Awakening: Predicting external stimulation to force transitions between different brain states
Source: Proc Natl Acad Sci U S A. 2019 Aug 19;116(36):18088–97. doi: 10.1073/pnas.1905534116 (PMC6731634; doi:10.1073/pnas.1905534116)
Supplement: Supplementary File [file pnas.1905534116.sapp.pdf]

Supplemental Information:

Awakening: predicting external stimulation to force transitions between different brain states

Gustavo Deco, Josephine Cruzat, Joana Cabral, Enzo Tagliazucchi, Helmut Laufs, Nikos K. Logothetis & Morten L. Kringelbach

Materials and methods

EEG-fMRI Recordings

From a total of fifty-five subjects (thirty-six females, mean±SD age of 23.4±3.3 years) who fell asleep during a simultaneous EEG-fMRI recording previously described in Tagliazucchi and Laufs (1), we selected the 18 subjects who reached stage N3 sleep (deep sleep). All subjects gave written informed consent with approval by the local ethics committee as described in Tagliazucchi and Laufs (1). The mean duration (± standard deviation) of contiguous N3 sleep epochs for these participants was 11.67 ± 8.66 minutes. The fMRI data was recorded at 3T (Siemens Trio, Erlangen, Germany) simultaneously with EEG data using an MR-compatible EEG cap (modified BrainCapMR, EasyCap, Herrsching, Germany). Sleep stages were scored manually by an expert according to the AASM criteria (2). fMRI data was realigned, normalized and spatially smoothed using SPM8 (www.fil.ion.ucl.ac.uk/spm). Cardiac, respiratory, and motion-induced noise were regressed out from the fMRI BOLD signals (3) and data was band-pass filtered in the range 0.01-0.1 Hz (4). Please see Tagliazucchi and Laufs (1) for full acquisition, pre-processing and sleep scoring details (5).

fMRI Pre-processing

For each participant and for each brain state (i.e. wakefulness and deep sleep), we used FSL tools to extract and average the BOLD signals from all voxels within each ROI defined in the AAL atlas (considering only the 90 cortical and subcortical non-cerebellar brain regions) (6).

We computed the empirical FC using pairwise Pearson correlation between all 90 regions, resulting in a 90x90 functional connectivity (FC) matrix for each participant and brain state. Correlation values were converted to z-values applying Fisher's transform before averaging across participants in the same cohort, resulting in a 90x90 FC matrix for each brain state (rest and sleep).

Structural Connectivity

In the whole-brain network model, the interactions between the 90 brain areas were scaled in proportion to their white matter structural connectivity. For the present study, we used the structural connectivity between the 90 AAL regions obtained in a previous study (7), averaged across 16 healthy young adults (5 females, mean±SD age: 24.75±2.54). Briefly, for each subject, a 90x90 structural connectivity matrix C was obtained by applying tractography algorithms to Diffusion Tensor Imaging (DTI) following the same methodology described in Cabral, Kringelbach and Deco (8) where the connectivity C_{np} between regions n and p is calculated as the proportion of sampled fibres in all voxels in region n that reach any voxel in region p. Since DTI does not capture fiber directionality, C_{np} was defined as the average between C_{np} and C_{pn}. Averaging

across all 16 participants resulted in a structural connectivity matrix *C* representative of healthy young adults.

References

1. Tagliazucchi E & Laufs H (2014) Decoding wakefulness levels from typical fMRI resting-state data reveals reliable drifts between wakefulness and sleep. *Neuron* 82(3):695-708.
2. AASM (2007) *The AASM manual for the scoring of sleep and associated events-rules. Terminology and technical specifications* (American Academy of Sleep Medicine, Chicago).
3. Glover GH, Li TQ, & Ress D (2000) Image-based method for retrospective correction of physiological motion effects in fMRI: RETROICOR. *Magnetic resonance in medicine : official journal of the Society of Magnetic Resonance in Medicine / Society of Magnetic Resonance in Medicine* 44(1):162-167.
4. Cordes D*, et al.* (2001) Frequencies contributing to functional connectivity in the cerebral cortex in "resting-state" data. *AJNR. American journal of neuroradiology* 22(7):1326-1333.
5. Deco G*, et al.* (2017) Perturbation of whole-brain dynamics in silico reveals mechanistic differences between brain states. *Neuroimage* 169:46-56.
6. Tzourio-Mazoyer N*, et al.* (2002) Automated anatomical labeling of activations in SPM using a macroscopic anatomical parcellation of the MNI MRI single-subject brain. *NeuroImage* 15(1):273-289.
7. Deco G*, et al.* (2017) Single or multiple frequency generators in on-going brain activity: A mechanistic whole-brain model of empirical MEG data. *NeuroImage* 152:538-550.
8. Cabral J, Kringelbach ML, & Deco G (2012) Functional graph alterations in schizophrenia: a result from a global anatomic decoupling? *Pharmacopsychiatry* 45 Suppl 1:S57-64.
